# Dysregulated TIE‐2 expression is associated with blood‐brain barrier leakiness and Alzheimer's disease‐related neuropathology

**DOI:** 10.1111/bpa.70109

**Published:** 2026-06-07

**Authors:** Selvi Ince, Carol Van Hulle, Gwendlyn Kollmorgen, Clara Quijano‐Rubio, Carey Gleason, Barbara B. Bendlin, Ozioma Okonkwo, Sanjay Asthana, Kaj Blennow, Henrik Zetterberg, Sterling C. Johnson, Seth Love, J. Scott Miners

**Affiliations:** ^1^ Cerebrovascular & Dementia Research Group, Learning and Research Building Bristol Medical School, Translational Health Sciences, University of Bristol Bristol UK; ^2^ Department of Medicine University of Wisconsin Madison Wisconsin USA; ^3^ Wisconsin Alzheimer's Disease Research Center University of Wisconsin Madison Wisconsin USA; ^4^ Roche Diagnostics GmbH Penzberg Germany; ^5^ Roche Diagnostics International Ltd Rotkreuz Switzerland; ^6^ Wisconsin Alzheimer's Institute University of Wisconsin Madison Wisconsin USA; ^7^ Clinical Neurochemistry Laboratory Sahlgrenska University Hospital Mölndal Sweden; ^8^ Department of Neurodegenerative Disease UCL Institute of Neurology London UK; ^9^ Department of Psychiatry and Neurochemistry, Institute of Neuroscience and Physiology Sahlgrenska Academy at University of Gothenburg Mölndal Sweden; ^10^ UK Dementia Research Institute at UCL London UK; ^11^ Hong Kong Center for Neurodegenerative Diseases Clear Water Bay Hong Kong China; ^12^ Department of Pathology and Laboratory Medicine University of Wisconsin School of Medicine and Public Health Madison Wisconsin USA; ^13^ Centre for Brain Research Indian Institute of Science Bangalore India

**Keywords:** Alzheimer's disease, angiopoietin‐2, BBB, blood–brain barrier, mild‐cognitive impairment, TIE‐1, TIE‐2

## Abstract

Cerebral hypoperfusion and blood–brain barrier (BBB) leakiness are related to cognitive decline and the onset and spread of Aβ and tau pathology in Alzheimer's disease (AD). Disrupted angiopoietin/TIE (ANGPT/TIE) signalling causes neurovascular instability and BBB leakiness accelerating cognitive decline and disease pathology in mouse models of AD. To explore ANGPT/TIE signalling in human AD, we measured CSF TIE‐1 and TIE‐2 levels by ELISA in two independent clinical dementia cohorts, and serum and CSF Tie‐1/‐2 in paired CSF and serum samples from neurologically normal individuals. ANGPT1, ANGPT2, TIE‐1 and TIE‐2 levels were measured by ELISA in crude homogenate (CH) and microvessel‐enriched fractions (MVFs) of post‐mortem human parietal cortex in relation to biochemical markers of cerebral perfusion (MAG:PLP1) and BBB leakiness (parenchymal fibrinogen) in a control, AD, and vascular dementia (VaD) cohort. CSF soluble TIE‐2 was elevated in AD biomarker+ve individuals and correlated positively with CSF t‐tau and p‐tau, and markers of BBB leakiness, neuronal injury, and neuroinflammation. Tissue TIE‐2 levels were significantly reduced in Braak tangle stage (BS) III‐IV, that is, brains with early‐intermediate AD pathology, and were lower in MVFs in end‐stage AD pathology (BSV‐VI) than in controls or VaD. Lower levels of MVF TIE‐2 correlated with markers of cerebral hypoperfusion and BBB leakiness. Our study reveals a reciprocal relationship between elevated CSF and reduced tissue TIE‐2 expression that is related to markers of tau pathology, BBB leakiness, and cerebral hypoperfusion, providing novel insights into ANGPT/TIE signalling in AD.

## Graphical abstract

Ince et al., have analysed TIE‐1 and ‐2 expression in human brain tissue, including microvessel‐enriched fractions (MVF) and covering a spectrum of AD‐associated pathology, and in CSF from independent cohorts spanning a clinical spectrum from normal cognition to AD. TIE‐2 was reduced in brain parenchyma and vessels but elevated in CSF, and the levels were related to CSF tau and markers of cerebral hypoperfusion, BBB leakiness, neuroinflammation and neuronal injury, in keeping with experimental rodent studies implicating abnormal ANGPT‐TIE signalling and vascular dysfunction in Alzheimer's disease.

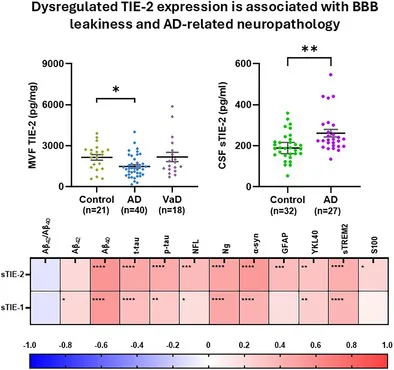

## 1 INTRODUCTION

Cerebrovascular pathology, including cerebral amyloid angiopathy (CAA) and cerebral small vessel disease (cSVD), are common in Alzheimer's disease (AD) [1]. Reduced whole‐brain and regional cerebral blood flow within the parietal cortex is associated with accelerated cognitive decline and the onset and spread of disease pathology in AD [2, 3, 4]. In autosomal dominant AD, reduced blood flow was evident in the precuneus up to 10 years prior to the predicted onset of clinical symptoms and mapped closely to the regional spread of PET‐Aβ [5]. A recent meta‐analysis confirmed the presence of cerebral hypoperfusion within the precuneus in sporadic AD [6]. White matter imaging abnormalities, including white matter hyperintensities which reflect cSVD, predict cognitive decline in AD [7] prior to the onset of clinical symptoms [8]. In familial AD, ischaemic white matter changes were found in the parietal and occipital lobe up to 22 years before the predicted onset of cognitive decline [9]. The deleterious effects of regional reductions in cerebral blood flow in AD are almost certainly mediated by impaired neurovascular coupling, which disturbs the fine balance between local blood flow and metabolic demand, and has been demonstrated in both sporadic AD and before the onset of cognitive decline in familial AD [10, 11].

Neurovascular stability depends on many signalling pathways including angiopoietin (ANGPT)/tyrosine kinase with immunoglobulin‐like and EGF‐like domains −1 and −2 (TIE‐1/‐2) signalling. ANGPT1, released by pericytes, acts upon endothelial TIE‐2 receptors, to mediate vascular stability [12, 13]. ANGPT2 serves as weak (decoy) agonist blocking endothelial TIE‐2 signalling and is associated with blood–brain barrier (BBB) breakdown [14]. In diabetic retinopathy, elevated expression of endothelial ANGPT2 activates TIE‐2 signalling in pericytes and is associated with pericyte degeneration [15, 16]. Adenoviral‐mediated endothelial overexpression of ANGPT2 accelerated cortical and hippocampal Aβ pathology in 5XFAD mice and was associated with BBB leakiness and neuroinflammation. In the same study, hippocampal Aβ plaque burden was reduced in 5xFAD mice crossed with endothelium‐specific *ANGPT2* KO mice. These protective effects were associated with reduced BBB leakiness and elevated phosphorylated TIE‐2 expression [17]. Analysis of single‐nuclei transcriptomic data of brain tissue from the ROSMAP study also revealed an enrichment of endothelial ANGPT2 in AD vs. MCI [17]. ANGPT2 levels, along with VEGF‐A, were elevated in post‐mortem vessel‐enriched preparations of parietal cortex in AD [18].

The *ANGPT2* gene has been consistently identified as one of the top‐ranking genes enriched in single‐nuclei transcriptomic analysis of purified vascular cells [19] and vessel‐enriched preparations [14] from human post‐mortem brain tissue in AD. In diseases that affect vascular permeability and angiogenesis, *ANGPT2* expression is dysregulated across numerous vascular cell‐types, including endothelial cells and smooth muscle cells [14]. The expression of a family of genes associated with neurovascular signalling, including VEGFR2, was reported to be dysregulated in AD [19]. Aβ‐induced ANGPT2 expression in cortical neurons of J20 mice highlights an important non‐vascular source of ANGPT2 that may contribute to pathogenic angiogenesis and vascular leakiness in AD [20].

We previously showed that CSF ANGPT2 levels were raised in MCI and AD, and correlated positively with CSF markers of disease pathology—Aβ_40_, t‐tau and p‐tau but not Aβ_42_. Elevated CSF ANGPT2 also correlated with markers of BBB leakiness—fibrinogen, albumin, sPDGFRβ and Qalb in paired CSF and serum samples; neuronal injury—increased neurogranin and α‐synuclein; and neuroinflammation, in AD [21]. In a recent study of matched serum:CSF samples from the ADNI study, CSF ANGPT2 levels correlated strongly with CSF t‐tau and p‐tau and cognitive decline in PET‐Aβ +ve mild‐cognitively impaired individuals [22]. CSF ANGPT2 levels were higher in archival post‐mortem CSF samples from donors with neuropathologically confirmed AD than with MCI in a small subset of samples from the Alzheimer's Disease Research Center (ADRC) [17]. These data suggest that ANGPT/TIE signalling is dysregulated in AD. However, despite a number of reports showing upregulated ANGPT2 expression in AD, it remains unclear whether TIE‐1 and TIE‐2 expression are similarly dysregulated.

In the present study, we have measured the levels in CSF of soluble TIE‐1 and TIE‐2 in three independent clinical cohorts comprising at‐risk cognitively unimpaired (CU) people, and people with mild cognitive impairment or AD, in relation to CSF markers of AD pathology, BBB leakiness, neuronal injury and neuroinflammation. We have also measured ANGPT1 and ANGPT2, and TIE‐1 and ‐2, in brain tissue and microvessel‐enriched fractions (MVFs) of parietal cortex (precuneus) in early‐ and late‐pathology AD, and explored whether altered expression of ANGPT and TIE proteins is associated with markers of cerebral hypoperfusion (reduced MAG:PLP1) and BBB leakiness (elevated brain tissue fibrinogen). This study builds upon recent findings in animal models and on human transcriptomic data to provide novel insights into ANGPT/TIE signalling, specifically characterising changes in TIE‐1 and TIE‐2 expression in human AD.

## 2 MATERIALS AND METHODS

### 2.1 CSF cohorts

CSF cohort (i) comprised lumbar CSF samples from the Clinical Neurochemistry Laboratory, Sahlgrenska University Hospital, Sweden. The samples were grouped as AD and non‐AD using established cut‐off values for previously measured levels of AD markers (INNOTEST, Fujirebio, Belgium): t‐tau >400 pg/mL, p‐tau >60 pg/mL and Aβ42 <550 pg/mL (all biomarkers positive for AD and all biomarkers negative for non‐AD) [23]. CSF cohort (ii) comprised lumbar puncture CSF samples from the Wisconsin Registry for Alzheimer's Prevention (WRAP) study and Wisconsin ADRC (WISC cohort). In this cohort, CSF samples were stratified into CU, mild cognitive impairment (MCI) and dementia‐AD. The diagnosis of MCI or dementia due to suspected AD was determined by a consensus review and was based on National Institute on Aging‐Alzheimer's Association criteria. CSF Aβ_1‐40_, Aβ _1–42_, t‐tau and p‐tau181 had previously been measured using the Roche NeuroToolKit, as described previously [24]. A panel of neuroinflammatory and neuronal injury markers had previously been measured [24]. Cohort (iii) comprised paired clinical CSF and serum samples from neurologically normal controls (21–86 years of age), provided by the Blennow/Zetterberg laboratories. The Qalb ratio had previously been determined using an immunoturbidimetric albumin method (Elecsys, Roche Diagnostics, Penzberg, Germany).

The demographic features of all three cohorts are summarised in Table 1. We were unable to measure all markers equally across the cohorts due to restrictions in CSF availability. The specific number of CSF samples assayed per marker is described within the figure legends. Clinical information related to *APOE* genotype and vascular co‐morbidities was not available for any of the cohorts.

**TABLE 1:** Summary of the clinical CSF and CSF/Serum cohorts.

	TIE‐1				TIE‐2			
*Clinical CSF cohort I (Sweden)*								
Diagnosis (*n*)	Control (32)	AD (34)			Control (32)	AD (27)		
Gender								
Female, *N* (%)	15 (42.9%)	14 (41.2%)		NS	13 (40.6%)	11 (40.7%)		NS
Male, *N* (%)	20 (57.1%)	20 (59.0%)			19 (59.4%)	16 (59.3%)		
Age at LP (year), mean (SD)	68.2 (12.4)	76.2 ± 6.1		<0.01	68.3 (12.4)	77.3 (5.5)		<0.001
*Clinical CSF cohort II (WRAP)*								
Diagnosis (*n*)	CU (82)	MCI (19)	AD (17)		CU (58)	MCI (15)	AD (14)	
Gender								
Female, *N* (%)	56 (68.3)	7 (36.8)	7 (41.2)		41 (70.7)	6 (40)	5 (35.7)	
Male, *N* (%)	26 (31.7)	12 (63.2)	10 (58.8)	<0.05	17 (29.3)	9 (60)	9 (64.3)	<0.05
Age at LP (years), mean (SD)	62.1 (5.8)	70.1 (6.5)	68.8 (5.9)	<0.0001	62.1 (6.2)	69.4 (7.1)	69.1 (6.2)	<0.0001
APOE ε4:								
APOE−, *N* (%)	47 (57.3%)	8 (42.1%)	3 (17.7%)		33 (56.9%)	7 (46.7%)	2 (14.3%)	
APOE+, *N* (%)	35 (42.7%)	11 (57.9%)	14 (82.4%)	<0.01	25 (43.1%)	8 (53.3%)	12 (85.7%)	<0.05
Amyloid status								
A−, *N* (%)	52 (63.41%)	3 (15.79%)	0 (0%)		39 (67.2%)	2 (13.3%)	0 (0%)	
A+, *N* (%)	27 (32.9%)	16 (84.2%)	17 (100%)	<0.0001	17 (29.3%)	13 (86.7%)	14 (100%)	0.0001
Tau status								
T−, *N* (%)	67 (81.7%)	5 (26.3%)	1 (5.9%)		48 (82.8%)	5 (33.3%)	0 (0%)	
T+, *N* (%)	12 (14.6%)	14 (73.7%)	16 (94.1%)	<0.0001	8 (13.8%)	10 (66.7%)	14 (100%)	<0.0001
*Serum/CSF cohort III (Sweden)*								
Sample type	CSF							
*N*	11				16			
Gender								
Female, *N* (%)	5 (45.5%)				8 (50%)			
Male, *N* (%)	6 (54.5%)				8 (50%)			
Age at LP, mean (SD)	63.45 (18.77)				58.56 (19.65)			
Sample type	Serum							
*N*	23				23			
Gender								
Female, *N* (%)	11 (47.8%)				11 (47.8%)			
Male, *N* (%)	12 (52.2%)				12 (52.2%)			
Age at Pb, mean (SD)	58.13 (20.25)				58.13 (20.25)			

Abbreviations: A, amyloid‐B; Age at Pb, age at phlebotomy; LP, lumbar puncture; n, Number; N, Percentage; SD, standard deviation; T, tau.

### 2.2 Brain tissue cohorts

Fresh frozen medial parietal cortex (Brodmann area 7) was provided by the South West Dementia Brain Bank (SWDBB) (University of Bristol, UK) with ethical approval. We studied samples from 21 control brain donors, 42 with AD and 18 with vascular dementia (VaD). AD donors had a clinical diagnosis of dementia with ‘intermediate’ or ‘high’ AD neuropathological change according to NIA‐AA guidelines [25]. Controls had no history of dementia and few/absent neuritic plaques, a Braak tangle stage of III or less, and no other major neuropathological abnormalities. VaD cases had a clinical history of dementia, multiple infarcts/ischaemic lesions, moderate to severe cerebral atheroma and/or arteriosclerosis, and only occasional neuritic plaques. For analysis of disease progression, the AD and control cohorts were combined and stratified into three subgroups according to Braak tangle stage (BS): BS0‐II, BSIII‐IV (intermediate pathology), BSV‐VI (late‐stage pathology). The demographic and neuropathological features of the cohorts used for brain tissue analysis are summarised in Table 2. A detailed breakdown of the demographic and neuropathological features within individual cases, including UK Brain Bank Network identifier numbers, is provided in Table S1.

**TABLE 2:** Summary of the post‐mortem cohorts.

(A)	BS0‐II	BSIII‐IV	BSV‐VI	*p* value
*N*	17	23	22	
Age at death (year), mean (SD)	84.5 (9.7)	85.0 (6.5)	78.3 (6.1)	<0.01
Gender				
Female, *N* (%)	7	11	10	NS
Male, *N* (%)	10	12	12	
Post‐mortem delay (h); mean (SD)	35.4 (14.7)	43.6 (24.6)	35.4 (22.9)	NS
Cognitive screening				—
CU	17	7	0	
CI	0	16	22	

(B)	Control	AD	VaD	*p* value
*N*	21	42	18	
Age at death (year); mean (SD)	84.5 (9.0)	81.4 (7.1)	81.5 (7.3)	NS
Gender				NS
Female, *N* (%)	8	20	8	
Male, *N* (%)	13	22	10	
Post‐mortem delay (h); mean (SD)	37.0 (15.3)	38.8 (24.3)	44.1 (19.9)	NS
Cognitive screening				—
CU	21	4	0	
CI	0	38	18	
Braak stage, *N*				—
BS0‐II	17	0	13	
BSIII‐IV	3	20	5	
BSV‐VI	0	22	0	

Abbreviations: BS, Braak tangle stage; CI, cognitively impaired; CU, cognitively unimpaired; N, number of donors; SD, standard deviation.

Brain tissue homogenates were prepared from 100 mg frozen parietal cortex homogenized at 20% w/v in 1% SDS lysis buffer (1% w/v SDS, 0.1 M NaCl, 0.01 M Tris–HCl (pH 7.6), 1 μM PMSF and 1 μg/mL of aprotinin) in a Precellys homogeniser (Stretton Scientific, Derbyshire, UK). Crude homogenate (CH) samples were sub‐aliquoted and stored at −80°C until required.

MVFs were prepared from 400 mg of frozen parietal cortex (precuneus) using a modified version of methods published by Lee [26] and Bourassa [27]. Brain tissue was rapidly minced on ice with a pre‐chilled scalpel in ice‐cold 2 mL PBS: 1% BSA and transferred to a 7 mL Dounce homogeniser. The tissue was homogenised with 20 initial strokes, followed by a further 15 strokes after the addition of 2 mL ice‐cold PBS:1% BSAand transferred to a 14 mL round‐bottom centrifuge tube and centrifuged at 2000*g* for 5 min at 4°C. The pellet was resuspended in 4 mL ice cold PBS:1% BSA and centrifuged at 2000*g* for 5 min at 4°C. The pellet was resuspended in 8 mL of 70 KDa molecular mass dextran (21% w/v) and the sample was mixed thoroughly and centrifuged at 4100*g* for 20 min with deceleration = 0. The (top) myelin layer and (underlying) supernatant were removed, and the pellet was resuspended in 1 mL 1xDPBS and passed through a 40 μm filter (Fisher Scientific) which was washed in ~10 mL DPBS to remove any debris adhered to the microvessels. To recover microvessels, the filter was inverted, placed on top of a new 50 mL falcon tube, and eluted with 10–20 mL of 1% BSA:DPBS. This was pelleted at 4100*g* for 10 min at 4°C, the supernatant was removed, and the pellet was resuspended in 1 mL of cold 1xPBS. After centrifugation at 12,000 rpm for 5 min, the pellet was lysed in 100 μL of CelLyticTM (Sigma Aldrich) lysis buffer containing Halt™ protease and phosphatase inhibitor cocktail (Thermo scientific, 1:100). Following centrifugation at 12,000*g* for 10 min, supernatants were collected and stored at −80°C.

Total proteins in crude and MVF homogenates were measured by Pierce™ Bradford plus protein assay (ThermoFisher Scientific).

### 2.3 ANGPT1 sandwich ELISA

ANGPT1 concentration was measured in duplicate in homogenates [1:80 dilution] of parietal cortex and in serum [1:120 dilution], using the human ANGPT1 DuoSet ELISA kit (R&D systems) as per the manufacturer's protocol. ANGPT1 levels in CSF and MVFs were below the threshold for detection. Absorbance was read at 450 nm in a FLUOstar OPTIMA reader (Labtech). Concentrations of ANGPT1 were interpolated from a standard curve generated using serial dilutions of recombinant human ANGPT1 (5000–39 pg/mL) supplied with the kit.

### 2.4 ANGPT2 sandwich ELISA

ANGPT2 concentration was measured in duplicate in CSF [1:2 dilution] and serum [1:5 dilution] using the human ANGPT2 Quantikine™ ELISA kit (R&D Systems). Brain homogenates [1:40 dilution] and MVFs [1:20 dilution] were measured in duplicate by human ANGPT2 DuoSet ELISA kit (R&D Systems). Absorbance was read at 450 nm in a FLUOstar OPTIMA plate reader (Labtech). Concentrations of ANGPT2 were interpolated from a standard curve generated using serial dilutions of recombinant human ANGPT2 (3000–23.5 pg/mL (Quantikine)) and (4000–62.5 pg/mL for CH and 4000–7.8 pg/mL for MVF [DuoSet]) supplied with the respective kits.

### 2.5 TIE‐1 sandwich ELISA

TIE‐1 concentration was measured in CSF [either neat or diluted 1:3 in single wells] and in serum [1:30 dilution], crude brain homogenates [1:10 dilution] and MVFs [1:11 dilution] in duplicate, with the human TIE‐1 DuoSet ELISA kit (R&D systems). Absorbance was read at 450 nm in a FLUOstar OPTIMA reader (Labtech). TIE‐1 concentrations were interpolated from a standard curve generated using serial dilutions of recombinant human TIE‐1 (5000–39 pg/mL for CH and 5000–9.8 pg/mL for MVF) supplied with the kit.

### 2.6 TIE‐2 sandwich ELISA

TIE‐2 concentration was measured in CSF [neat in a single well], and in duplicate in serum [1:50 dilution], crude brain homogenates and MVFs [both 1:40 dilution] with the human TIE‐2 DuoSet ELISA kit (R&D systems). Absorbance was read at 450 nm in a FLUOstar OPTIMA reader (Labtech). Concentrations of TIE‐2 were interpolated from a standard curve generated using serial dilutions of recombinant human TIE‐2 (5000–39 pg/mL for CH or 5000–9.8 pg/mL for MVF) supplied with the kit.

### 2.7 MAG and PLP1 ELISAs

Concentrations of MAG [diluted 1:60], by in‐house direct ELISA, and PLP1[diluted 1:80], by commercially available sandwich ELISA (SEA417Hu, USCN, Wuhan, China), were measured in brain tissue homogenates as previously described [28, 29, 30]. The concentrations of MAG and PLP1 were adjusted by the dilution factor prior to calculating the MAG:PLP1 ratio.

The MAG:PLP1 ratio is a post‐mortem marker used to assess ante‐mortem brain tissue oxygenation prior to death. Both myelin proteins are stable for up to 72 h post‐mortem and have similar slow turnover rates in vivo, of several months [28]. However, the two proteins are differentially affected by reduced oxygenation: MAG degrades more rapidly in response to chronic tissue hypoxia than does PLP1, possibly because transportation of MAG from the oligodendrocyte cell body to its location within the adaxonal myelin loop requires more energy than does the insertion of PLP, which is expressed throughout the myelin‐sheath. A reduction in MAG:PLP1 is a marker of chronic cerebral hypoperfusion (reviewed [31]), as demonstrated in multiple independent studies [28, 29, 30, 32, 33, 34].

### 2.8 Fibrinogen sandwich ELISA

Fibrinogen concentration in brain tissue homogenates was measured by commercially available sandwich ELISA (FineTest EH3057 kit, Wuhan Fine Biotech Co., China). CHs [diluted 1:600] were assayed in duplicate. The fibrinogen concentration was interpolated from a standard curve generated by serial dilution of human recombinant fibrinogen (100–1.6 ng/mL) supplied with the kit. Fibrinogen levels were adjusted to total protein levels.

### 2.9 CD31 sandwich ELISA

CD31 concentration, as a marker of vessel density, was measured in CH and MVFs, both diluted 1:60 in PBS, with a commercial DuoSet ELISA kit (DY806‐05, R&D systems). The concentration of CD31 for each case was interpolated from serial dilutions of recombinant human CD31 (10,000–156 pg/mL for CH or 10,000–78 pg/mL for MVF) supplied with the kit.

### 2.10 Statistical analysis

The distribution of each dataset was tested for normality by visual inspection and applying the D'Agostino & Pearson test. Normally distributed data, before or following log_10_ transformation, were analysed by one‐way ANOVA followed by Tukey's multiple comparison post hoc test. If the data were not normally distributed, even after log transformation, a Kruskal‐Wallis test was used to test the null hypothesis and Dunn's post hoc test was used to compare the subgroups within each cohort. Pearson's (or the non‐parametric equivalent) was used for correlation analysis.

## 3 RESULTS

### 3.1 CSF sTIE‐2 is elevated in AD and associated with CSF tau and BBB leakiness

Cohort (i) comprised clinical CSF lumbar samples stratified according to established CSF Aβ and tau cut‐off values [21] into controls and AD. CSF soluble TIE‐2 (sTIE‐2) was significantly higher in AD and correlated positively with CSF t‐tau (*r* = 0.47, *p* = 0.0002) and p‐tau181 (*r* = 0.49, *p* < 0.0001) but not with CSF Aβ_1‐42_ (Figure 1A). CSF sTIE‐2 mirrored previously reported changes in ANGPT2 level in the same cohort [21]. CSF soluble TIE‐1 (sTIE‐1) was unchanged in AD and correlated weakly with CSF p‐tau181 (*r* = 0.33, *p* = 0.0069) but not with CSF t‐tau or Aβ_1‐42_ (Figure 1B). ANGPT‐1 was below the threshold level for detection in CSF.

**FIGURE 1:**
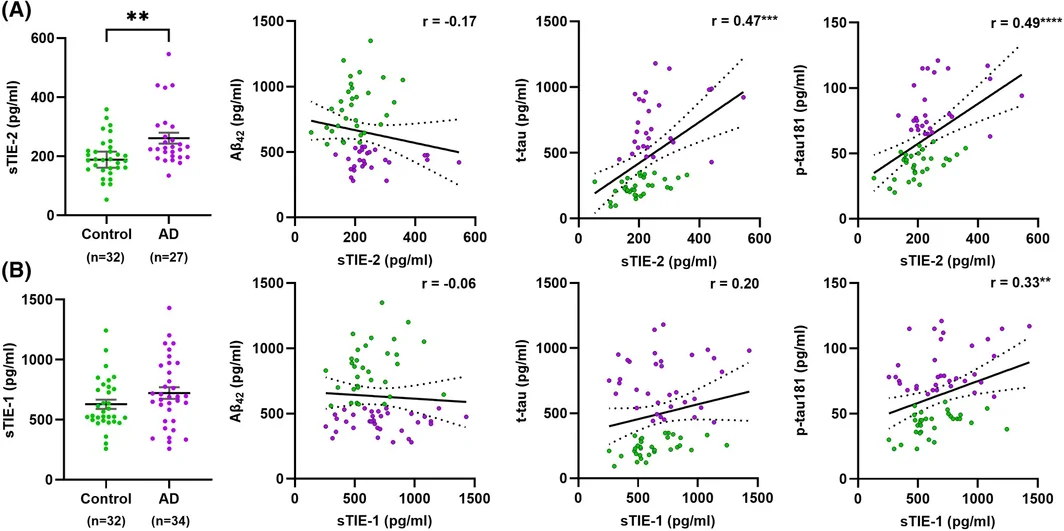
Elevated CSF TIE‐2 in Alzheimer's disease is associated with CSF t‐tau and p‐tau. Analysis of data from Cohort (i) shown in (A). Scatterplots showing CSF soluble TIE‐2 (sTIE‐2) level in age‐matched controls (*n* = 32) and AD cases (*n* = 27) (means ± SEM) in relation to CSF Aβ_1‐42_, t‐tau and p‐tau181 and (B). Scatterplots showing CSF levels of soluble TIE‐1 (sTIE‐1) in age‐matched controls (*n* = 32) and AD cases (*n* = 34) (means ± SEM) in relation to CSF Aβ_1‐42_, t‐tau and p‐tau181. Linear regression lines (solid) and 95% confidence interval lines (dashed) are shown. Each dot represents an individual case (green = control, purple = AD). Pearson correlation coefficients (*r*) are shown. **p < 0.01; ***p < 0.001; ****p < 0.0001.

CSF sTIE‐2 correlated positively but weakly with markers of BBB leakiness, including levels of albumin (*r* = 0.27 and *p* = 0.046), fibrinogen (*r* = 0.26 and *p* = 0.048) and IgG (*r* = 0.21, *p* = 0.11) (Figure 2A). CSF sTIE‐2 correlated strongly with sPDGFRβ (*r* = 0.56, *p* < 0.0001) (Figure 2A). CSF sTIE‐1 did not correlate with any of the three BBB markers and correlated only weakly with sPDGFRβ (*r* = 0.31, *p* = 0.012) (Figure 2B).

**FIGURE 2:**
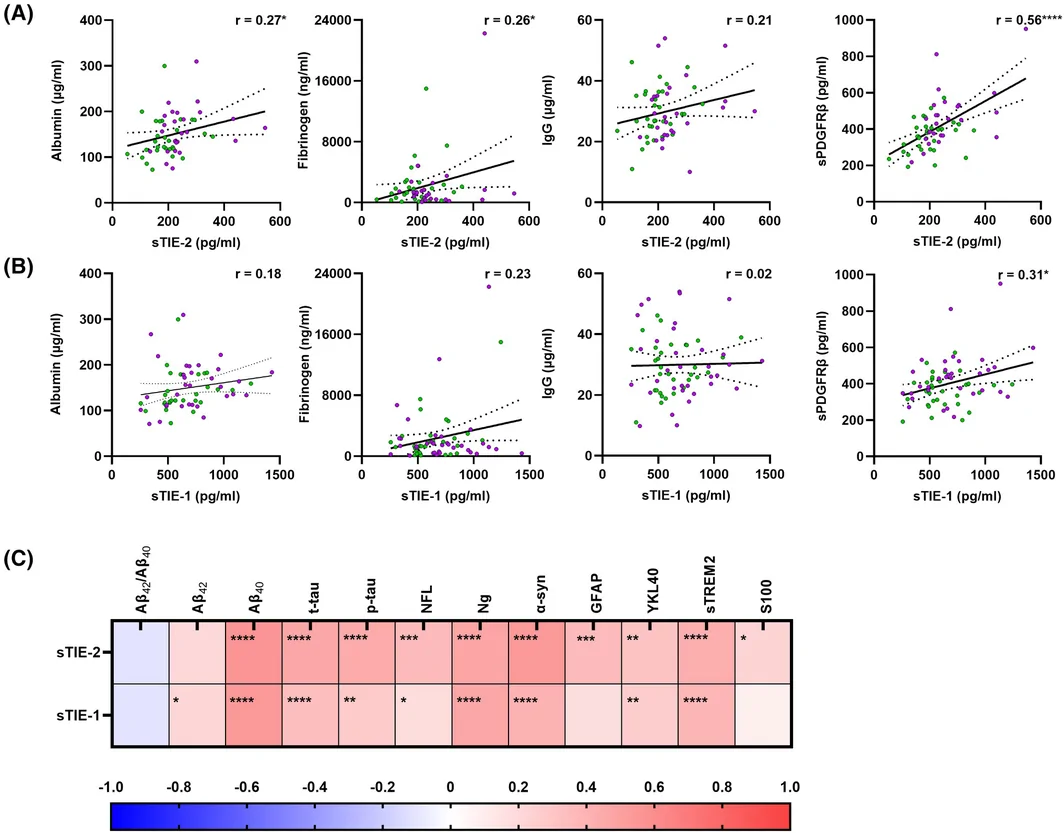
CSF TIE‐2 is related to markers of BBB leakiness, disease pathology, neuronal injury and inflammation. Analysis of data from Cohort (i) is shown in (A) and (B). Relationship of CSF sTIE‐2 and sTIE‐1 levels to CSF markers of BBB leakiness: Albumin, fibrinogen, IgG, and sPDGFRβ are shown. Linear regression lines (solid) and 95% confidence interval lines (dashed) are shown. Pearson's correlation co‐efficients (r) are displayed. Each dot represents the mean value from one case (green = controls, purple = AD). Analysis of data from Cohort (ii) is shown in (C). Heatmap summarising correlations between CSF sTIE‐1 and sTIE‐2 with markers of AD pathology, neuronal injury and neuroinflammation. *p < 0.05; **p < 0.01; ***p < 0.001; ****p < 0.0001.

Cohort (ii) comprised clinical CSF lumbar samples enriched for CU individuals (*n* = 82), with a small number of MCI (*n* = 19) and AD cases (*n* = 17) for comparison. CSF sTIE‐2 did not differ between diagnosis groups: CU (305 ± 85 pg/mL, *n* = 58), MCI (309 ± 111 pg/mL, *n* = 15), AD (310 ± 126 pg/mL, *n* = 14). Nor did CSF sTIE‐1: CU (641 ± 296 pg/mL, *n* = 82), MCI (674 ± 382 pg/mL, *n* = 19), AD (657 ± 179 pg/mL, *n* = 17). However, CSF sTIE‐1 and sTIE‐2 correlated strongly with a panel of CSF markers of AD neuropathological change (ADNC), neuroinflammation and neuronal injury, summarised in the heatmap (Figure 2C). Within the entire cohort, sTIE‐1 and sTIE‐2 correlated positively with Aβ_1‐40_, t‐tau and p‐tau181, as well as markers of neuronal injury (NFL, neurogranin (Ng), and ⍺‐syn) and neuroinflammation (GFAP, YKL40, sTREM2). When stratified into individual diagnosis groups, the relationships were strongest in CU and MCI, and weak or absent in AD (Figure S1). CSF sTIE‐1 and sTIE‐2 levels also correlated positively with each other and with ANGPT2 (Figure S2).

To further explore the relationship between CSF sTIE‐1 and sTIE‐2 and BBB leakiness, we performed correlation analysis in 23 matched CSF and serum samples (cohort (iii)), in which the CSF/serum albumin ratio (Qalb) had previously been measured. CSF sTIE‐2 correlated positively and strongly with Qalb (*p* = 0.72, *p* = 0.0018) (Figure 3A) whereas CSF sTIE‐1 did not (Figure 3B). Serum sTIE‐1 (*r* = 0.31, *p* = 0.161) and serum sTIE‐2 (*r* = 0.38, *p* = 0.07) showed a positive trend with Qalb approaching significance (Figure 3C,D)but were unrelated to respective CSF sTIE‐1 and sTie‐2 levels (Figure 3E,F).

**FIGURE 3:**
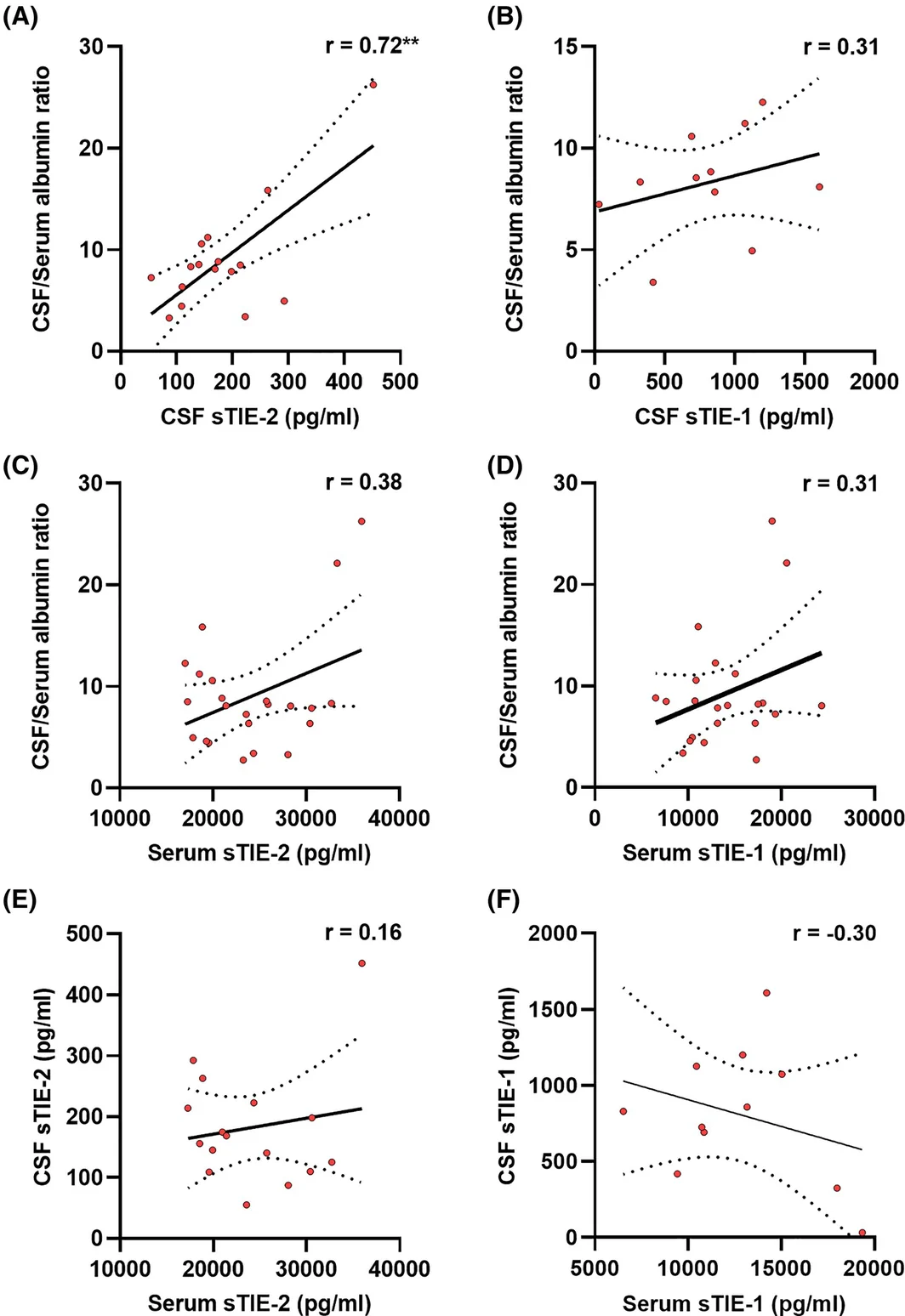
CSF TIE‐2 correlates with the BBB marker Qalb in neurologically normal controls. (A,B) Scatterplots showing relationship of CSF sTIE‐1 and sTIE‐2 with Qalb—CSF/Serum albumin ratio. (C,D) Scatterplots showing relationship of serum sTIE‐1 and sTIE‐2 with Qalb. (E,F) Scatterplots showing relationship between CSF and serum for sTIE‐2 and sTIE‐1. Linear regression lines (solid) and 95% confidence interval lines (dashed) are shown. Pearson's correlation co‐efficient (r) is displayed. Each dot represents an individual case. **p < 0.01.

### 3.2 TIE‐2 level is lower in crude and microvessel‐enriched fractions of parietal cortex in AD and associated with markers of cerebral hypoperfusion and BBB leakiness

We measured ANGPT1/2 and TIE‐1/−2 protein level in crude brain homogenates (CH) and microvessel‐enriched fractions (MVFs) of parietal cortex. To assess how ANGPT2 and TIE expression vary with progression of AD, we first stratified the combined control and AD cohorts into Braak tangle stage subgroups: BS0‐II, III‐IV and V‐VI (Figure 4).

**FIGURE 4:**
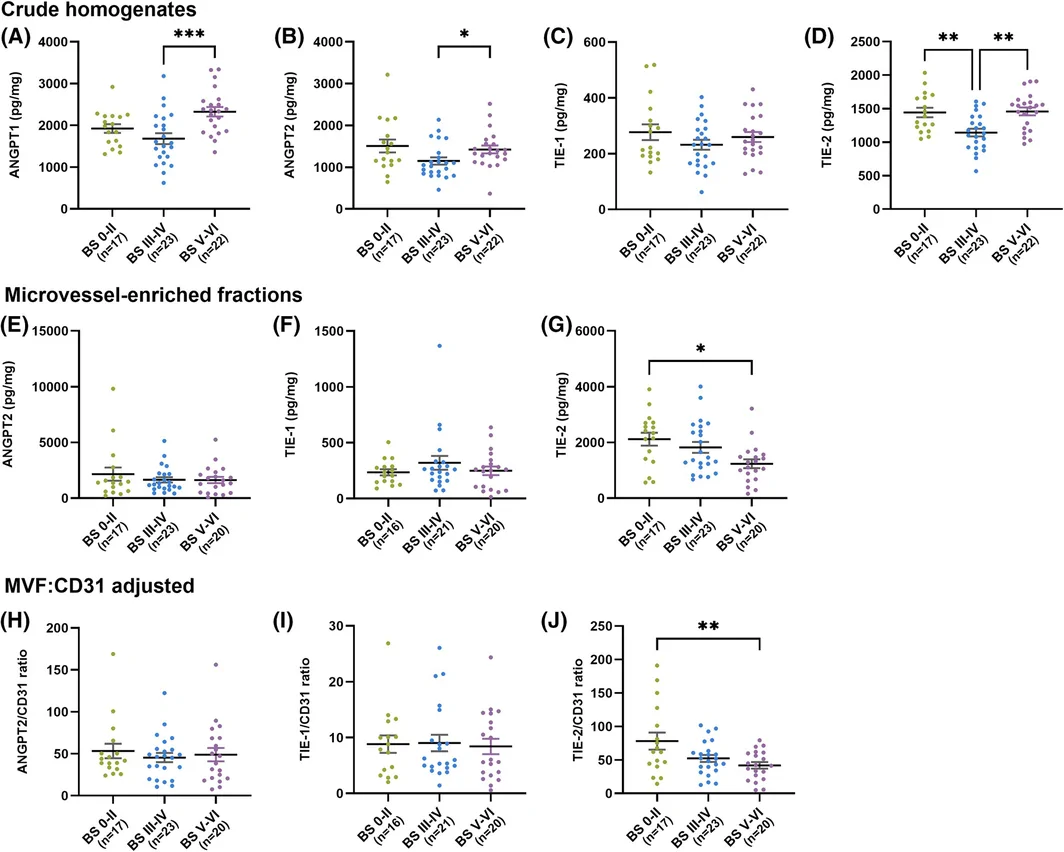
Comparison of ANGPT and TIE protein levels in crude homogenate (CH) and microvessel‐enriched fraction (MVF) preparations of parietal cortex (precuneus) between Braak tangle stage (BS) subgroups. (A–D) Scatterplots showing CH ANGPT1, ANGPT2, TIE‐1 and TIE‐2 levels in the BS subgroups. (E–H) Scatterplots showing MVF ANGPT2, TIE‐1 and TIE‐2 levels in the BS subgroups. (H–J) Scatterplots showing MVF ANGPT2, TIE‐1 and TIE‐2 levels adjusted for CD31 in relation to BS. Horizontal bars show mean ± SEM. Number of cases in each group shown in parenthesis. Each dot represents an individual case. *p < 0.05; **p < 0.01.

ANGPT1 (*p* < 0.001) and ANGPT2 (*p* < 0.05) levels within CHs were both significantly lower in BSIII‐IV than BSV‐VI (Figure 4A,B). TIE‐1 was unchanged across Braak stage groups (Figure 4C); however, TIE‐2 level was significantly lower in BSIII‐IV than BS0‐II, and BSV‐VI (*p* < 0.01 for both) (Figure 4D).

ANGPT2 in MVFs did not vary with Braak stage (Figure 4E), and ANGPT1 was undetectable. MVF TIE‐1 also did not vary with Braak stage (Figure 4F), however, MVF TIE‐2 level was lower in BSV‐VI than BS0‐II (*p* < 0.05) (Figure 4G). Adjustment for CD31, to account for potential variation in vessel density between cases, did not alter the findings with respect to MVF ANGPT2 or TIE‐1 (Figure 4H,I) but increased the significance of the difference in MVF TIE‐2 between BS0‐II and BSV‐VI (*p* < 0.01) (Figure 4J). Absolute CD31 levels were unaltered in relation to Braak tangle stage (Figure S3A).

We also compared ANGPT and TIE protein levels in both CH and MVFs between control, AD and VaD cases (Figure 5). CH ANGPT1 and ANGPT2, and CH TIE‐1 and ‐2 did not differ significantly between AD, VaD and controls (Figure 5A–D). MVF ANGPT2 level was lower in AD than VaD (*p* < 0.05) (Figure 5E). MVF TIE‐2 was significantly lower in AD than controls (p < 0.05) (Figure 5G). Adjustment for CD31 did not alter the findings with respect to MVF ANGPT2 or TIE‐1 (Figure 5H,I) but increased the significance of the difference in MVF TIE‐2 between AD and controls (*p* < 0.01) (Figure 5J). CD31 level did not vary in relation to diagnosis group (Figure S3B).

**FIGURE 5:**
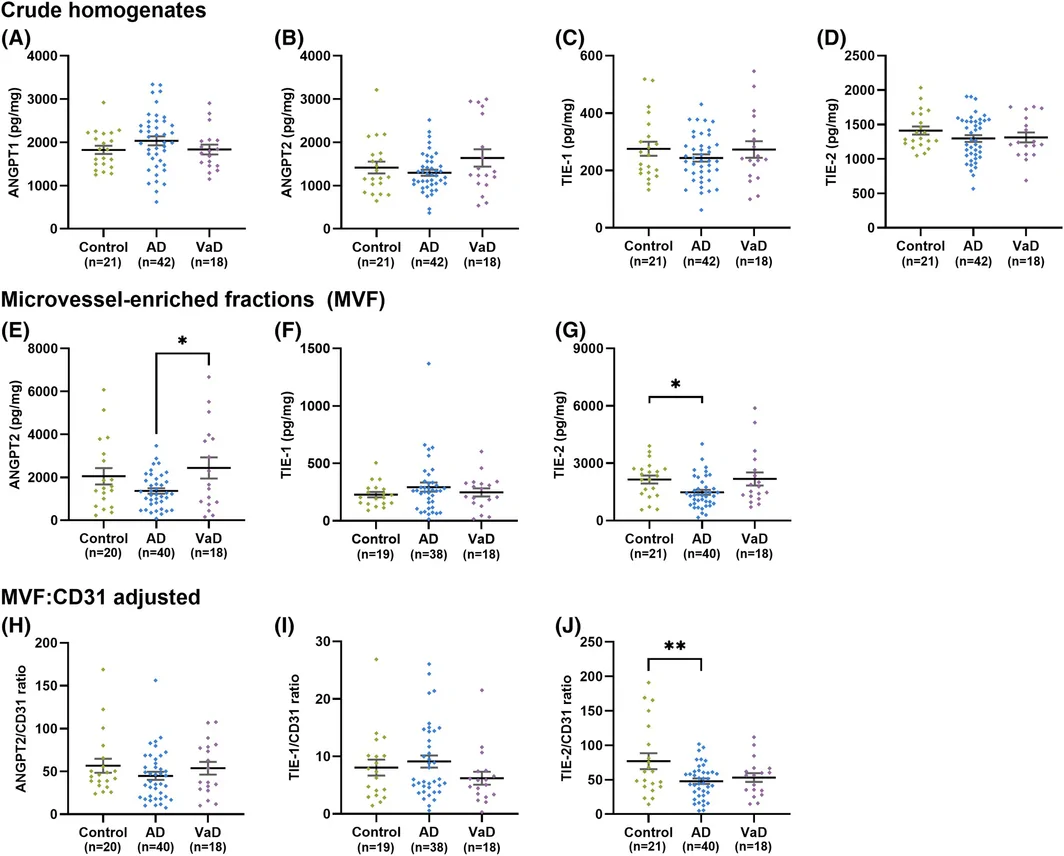
Comparison of ANGPT and TIE protein levels in CH and MVF preparations of parietal cortex (precuneus) between controls, AD and VaD. (A–D) Scatterplots showing CH ANGPT1, ANGPT2, TIE‐1 and TIE‐2 levels in controls, AD and VaD cases. (E–G) Scatterplots showing MVF ANGPT2, TIE‐1 and TIE‐2 in each group (H–J). Scatterplots showing ANGPT2, TIE‐1 and TIE‐2 adjusted for CD31 in each group. Horizontal bars show mean ± SEM. Each dot represents an individual case. *p < 0.05; **p < 0.01.

To examine the relationship of the ANGPT and TIE proteins to markers of cerebrovascular function across the full spectrum of AD pathology, we combined the control and AD cohorts for correlation analysis. MAG:PLP1 was lower in BSV‐VI than in BS0‐II or BSIII‐IV (*p* < 0.0001 for both) and was lower in AD compared to controls or VaD (*p* = 0.087 and *p* = 0.028, respectively) (Figure S4A,C). MAG:PLP1 correlated negatively with CH ANGPT‐1 (*r* = −0.25, *p* = 0.047) and positively with MVF TIE‐2 (*r* = 0.35, *p* = 0.007) (Figure 6A,G). Fibrinogen level, a marker of BBB leakiness, was higher in BSV‐VI than BSIII‐IV but did not differ significantly between AD, VaD and controls (Figure S4B,D). In the combined cohort, fibrinogen level correlated positively with CH ANGPT‐1 (*r* = 0.31, *p* = 0.013) and ANGPT2 (*r* = 0.32, *p* = 0.012) and negatively with MVF TIE‐2 (*r* = −0.28, *p* = 0.031) (Figure 7A,B,G). No other correlations were observed.

**FIGURE 6:**
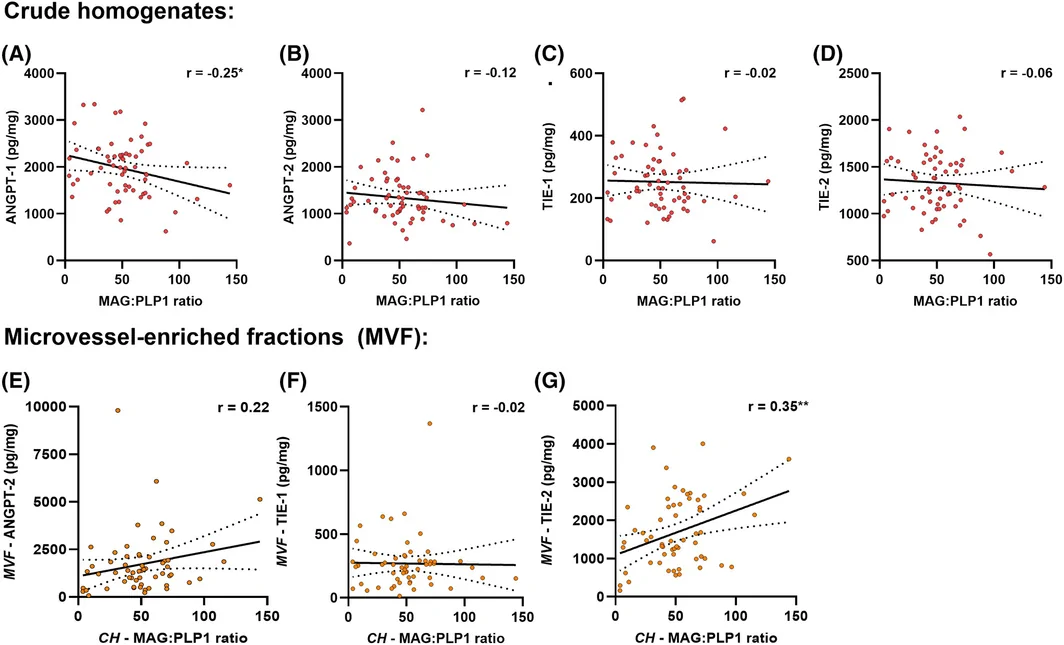
ANGPT/TIE expression is related to a marker of cerebral perfusion (MAG:PLP1) in the parietal cortex. (A–D) Correlations between ANGPT1, ANGPT2, TIE‐1 and TIE‐2 levels in crude homogenates in relation to MAG:PLP1 within combined AD and controls. (E–G) Correlations between MVF levels of ANGPT‐1, ANGPT2, TIE‐1 and TIE‐2 and MAG:PLP1 within combined AD and controls. Linear regression line (solid) and 95% confidence interval lines (dashed) are shown. Pearson's correlation co‐efficients (*r*) are displayed. Each dot represents an individual case. *p < 0.05; **p < 0.01.

**FIGURE 7:**
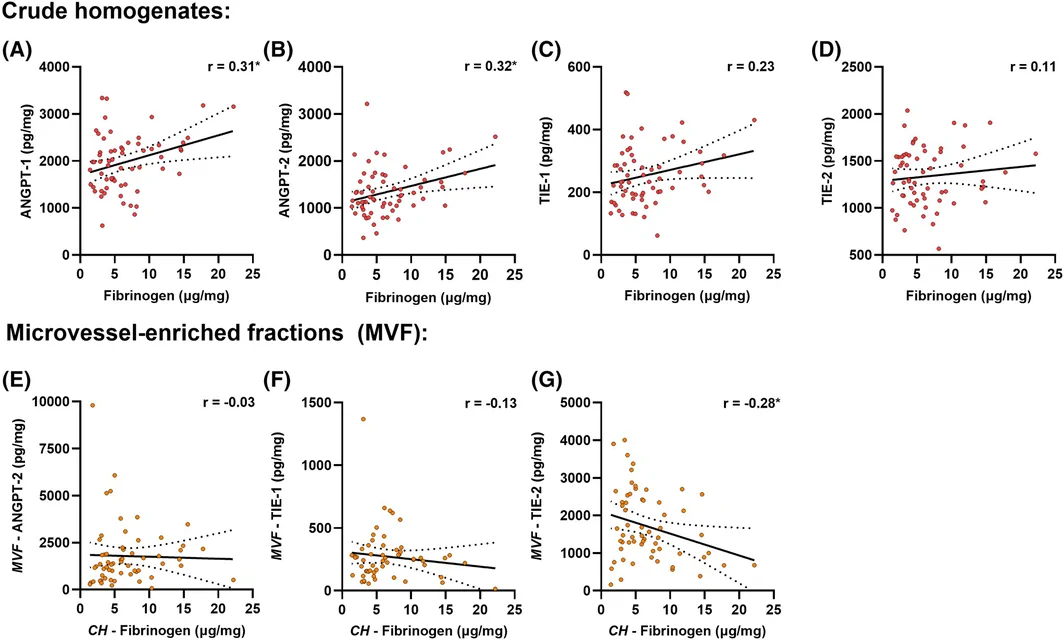
ANGPT/TIE expression is related to fibrinogen, a marker of BBB breakdown, in the parietal cortex. (A–D) Correlations between ANGPT‐1, ANGPT2, TIE‐1 and TIE‐2 levels in crude homogenates in relation to fibrinogen levels in combined AD and controls. (E–G) Correlations shown for MVF levels of ANGPT‐1, ANGPT2, TIE‐1 and TIE‐2 and fibrinogen in combined AD and controls. Linear regression line (solid) and 95% confidence interval lines (dashed) are shown. Pearson's correlation co‐efficients (*r*) are shown. Each dot represents an individual case *p < 0.05.

## 4 DISCUSSION

In the present study, we have analysed TIE‐1/‐2 levels in CSF and ANGPT1/2 and TIE‐1/‐2 in post‐mortem crude brain tissue and MVFs in the parietal cortex in AD. The findings build upon recent studies showing elevated endothelial ANGPT2 expression associated with cognitive decline and Aβ pathology as a result of BBB breakdown and neuroinflammation in mouse models of AD [17]. Endothelial ANGPT2 gene and protein expression are also elevated in human post‐mortem tissue [17, 18, 19, 35] and CSF ANGPT2 levels are increased in AD [21]. We have added to this data, revealing that TIE‐2 expression is also altered in AD: CSF sTIE‐2 levels were higher in AD and correlated with CSF markers of tau pathology and BBB leakiness (reflecting previous findings for ANGPT2 [21]) but TIE‐2 levels were correspondingly lower in CH and MVFs of parietal cortex. The reduction in MVF TIE‐2 correlated with markers of cerebral hypoperfusion (lower MAG:PLP1) and BBB leakiness (elevated fibrinogen). We propose that these findings potentially reflect increased shedding of membrane‐bound TIE‐2 upon endothelial injury (as seen for other markers of neurovascular instability such as soluble PDGFRβ [36]). The study reveals that ANGPT/TIE signalling is dysregulated in AD, associated with lower protective TIE‐2 tissue expression and elevated CSF soluble TIE‐2, and is closely related to markers of BBB leakiness and tau pathology, detectable even before established AD (mirroring previous findings for ANGPT2).

CSF markers of endothelial and pericyte injury are elevated in AD, particularly in MCI [22, 37, 38]. Levels of endothelial injury markers, including VCAM‐1, ICAM‐2 and VEGFR1, were found to increase in MCI and to correlate strongly with t‐tau and p‐tau, cortical thinning and cognitive decline, particularly in Aβ_1‐42_ positive individuals [37]. Similar findings were recently reported for CSF sPDGFRβ, a marker of pericyte injury, which was strongly related to CSF t‐tau and p‐tau in Aβ_1‐42_ positive individuals and levels were elevated during MCI‐AD transition, associated with CSF markers of neuronal injury and neuroinflammation [36, 38, 39, 40]. Recent studies provide evidence that neurovascular disruption may precede BBB leakiness in at‐risk cognitively unimpaired (CU)  individuals. We reported that CSF Placental Growth Factor (PlGF) correlated strongly with CSF tau and cognitive decline in PET‐Aβ +ve individuals prior to BBB leakiness [22]. When the cohort was stratified according to diagnosis (CU, MCI and AD), the relationship between markers of neurovascular injury, including ANGPT2, with markers of tau and cognitive decline, was strongest in CU and MCI [22], supporting our earlier observations [21]. CSF sPDGFRβ levels correlated strongly with an age‐ and disease‐related increase in hippocampal BBB leakiness identified by high contrast MRI imaging [41, 42]. In a large study of the Swedish BioFINDER 2 cohort, CSF sPDGFRβ increased with age and correlated with Qalb [43]. CSF ANGPT2 correlated positively with Qalb in paired CSF and serum samples from neurologically normal controls [21]. In the present study, CSF sTIE‐2 levels also correlated strongly with CSF t‐tau and p‐tau, and with Qalb.

Elevated CSF sTIE‐2 level, correlated strongly with markers of neuronal injury (YKL‐40, Ng, NFL) and neuroinflammation (GFAP, TREM‐2), reflecting similar observations for ANGPT2 [21]. As for ANGPT2, these relationships were strongest in the cognitively unimpaired and MCI groups. These findings mirror associations with markers of neuronal injury markers and inflammation observed for endothelial (ICAM‐1, VCAM‐1, Flt1) [37] and pericyte (sPDGFRβ) [39, 43] markers and together highlight a close relationship between cerebrovascular dysfunction, neuroinflammation, and neuronal and synaptic remodelling starting in early‐stage AD. Serum sTIE‐2 correlated weakly with Qalb, as did serum ANGPT2; a possible relationship between serum ANGPT/TIE signalling markers and BBB leakiness need to be confirmed in larger independent cohorts. Longitudinal studies are also required to clarify the temporal relationships between these markers of different pathological processes, and to fully investigate their diagnostic potential.

To the best of our knowledge, the expression of ANGPT/TIE signalling proteins within human brain tissue, and their relationship to markers of cerebral blood flow and BBB permeability, have not previously been fully characterised. Since ANGPT/TIE proteins are expressed in both vascular and non‐vascular cells within the brain, we analysed both crude brain homogenates (CH) and microvessel‐enriched fractions (MVFs) and compared the findings to those in CSF to gain insight into possible mechanisms underlying disease‐related changes in the ANGPT/TIE signalling pathway. TIE2 in the MVF showed a non‐significant reduction in BSIII‐IV but a highly significant reduction in BSV‐VI. In CHs, ANGPT‐1, ANGPT2 and TIE‐2 were all lowest in BSIII‐IV (and levels in BSV‐VI were similar to controls). Proteolytic cleavage of membrane‐bound TIE‐2 from injured endothelial cells was reported previously [44, 45] and might explain the reciprocal relationship observed between lower TIE‐2 in brain tissue and higher levels in CSF. However, it is also possible that TIE‐2 in CSF may originate partly from non‐vascular sources, including neurons [46] and glial cells [47]. Neuronal expression of angiogenic peptides, including ANGPT2, was shown to be upregulated in response to Aβ peptides [20] – a non‐vascular source may therefore also contribute to elevated CSF TIE‐2 in AD.

The present study has limitations. Although the CSF findings were replicated across two independent clinical cohorts, both cohorts were relatively small. The cross‐sectional nature of the study precluded an analysis of temporal influences on causality, and the impact of potential confounders, such as vascular co‐morbidities and *APOE* genotype, could not be assessed. The finding of a relationship between CSF and serum TIE‐2 with Qalb was performed in matched CSF:serum samples for only a small cohort (*n* = 23) of neurologically normal controls. The potential of serum or plasma markers of TIE2 as surrogates for BBB leakiness and ADNC requires validation in a larger study with the inclusion of samples from disease‐relevant participants. The biochemical assessment of post‐mortem brain tissue and MVFs allowed us to explore the relationships between ANGPT/TIE signalling and markers of brain perfusion and BBB integrity but not to analyse these relationships mechanistically or at a cellular level; such analysis will need further studies.

In conclusion, we have presented evidence of abnormalities of the ANGPT/TIE signalling pathway in AD—specifically, elevated CSF sTIE‐2 and concurrent tissue TIE‐2 loss within the parietal cortex, associated with markers of BBB damage, hypoperfusion and tau pathology. These abnormalities are detectable with early‐stage AD pathology, and the CSF changes precede clinical AD diagnosis.

## AUTHOR CONTRIBUTIONS

SI, SL and JSM devised the original study and experimental plan. SI generated, collected and analysed the data. CVH performed additional analysis. All authors reviewed the manuscript and SL and JSM prepared it for publication.

## FUNDING INFORMATION

The Wisconsin Cohort is supported by the following grants: NIH R01AG027161, P50AG033514, R01AG054059, R01 AG062167, UF1AG051216. Support was also provided by the NIH National Center for Advancing Translational Sciences (NCATS), grant UL1TR000427 (Clinical and Translational Sciences). SI is supported under scholarship programme called YLSY by Ministry of National Education in Türkiye. JSM is supported by an ARUK Senior Fellowship award (ARUK‐SRF‐2019A‐001). COBAS and ELECSYS are trademarks of Roche. All other product names and trademarks are property of their respective owners. The NeuroToolKit is a panel of exploratory prototype assays designed to robustly evaluate biomarkers associated with key pathologic events characteristic of AD and other neurological disorders, used for research purposes only and not approved for clinical use (Roche Diagnostics International Ltd., Rotkreuz, Switzerland). Elecsys β‐amyloid (1–42) CSF and Elecsys Phospho‐Tau (181P) CSF assays are approved for clinical use. HZ is a Wallenberg Scholar and a Distinguished Professor at the Swedish Research Council supported by grants from the Swedish Research Council (#2023‐00356, #2022‐01018 and #2019‐02397), the European Union's Horizon Europe research and innovation programme under grant agreement No 101053962, and Swedish State Support for Clinical Research (#ALFGBG‐71320).

## CONFLICT OF INTEREST STATEMENT

HZ has served at scientific advisory boards and/or as a consultant for Abbvie, Acumen, Alector, Alzinova, ALZpath, Amylyx, Annexon, Apellis, Artery Therapeutics, AZTherapies, Cognito Therapeutics, CogRx, Denali, Eisai, Enigma, LabCorp, Merck Sharp & Dohme, Merry Life, Nervgen, Novo Nordisk, Optoceutics, Passage Bio, Pinteon Therapeutics, Prothena, Quanterix, Red Abbey Labs, reMYND, Roche, Samumed, ScandiBio Therapeutics AB, Siemens Healthineers, Triplet Therapeutics, and Wave, has given lectures sponsored by Alzecure, BioArctic, Biogen, Cellectricon, Fujirebio, LabCorp, Lilly, Novo Nordisk, Oy Medix Biochemica AB, Roche, and WebMD, is a co‐founder of Brain Biomarker Solutions in Gothenburg AB (BBS), which is a part of the GU Ventures Incubator Program, and is a shareholder of CERimmune Therapeutics (outside submitted work). KB has served as a consultant and at advisory boards for Abbvie, AC Immune, ALZPath, AriBio, Beckman‐Coulter, BioArctic, Biogen, Eisai, Lilly, Moleac Pte. Ltd., Neurimmune, Novartis, Ono Pharma, Prothena, Quanterix, Roche Diagnostics, Sunbird Bio, Sanofi and Siemens Healthineers; has served at data monitoring committees for Julius Clinical and Novartis; has given lectures, produced educational materials and participated in educational programmes for AC Immune, Biogen, Celdara Medical, Eisai and Roche Diagnostics; and is a co‐founder of Brain Biomarker Solutions in Gothenburg AB (BBS), which is a part of the GU Ventures Incubator Program, outside the work presented in this paper. The other authors report no disclosures.

## ETHICS STATEMENT

The study was approved by the Southwest Dementia Brain Bank (SWDBB) management committee (Human Tissue Authority licence number 12273) under the terms approved by the Bristol (18/SW/0029) ethical research committee. All WRAP and WADRC protocols were approved by the University of Wisconsin‐Madison institutional review board.

## Supporting information


**Figure S1.** Heatmaps showing correlations between CSF TIE‐1 and TIE‐2 in relation to markers of ADNC, neuronal injury, and neuroinflammation. Data from Cohort (ii).
**Figure S2.** Heatmap showing correlations between sTIE‐1, sTIE‐2, and ANGPT2. Data from Cohort (ii).
**Figure S3.** MVF CD31 levels are unaltered in relation to Braak tangle stage (BS) and do not differ in AD or VaD compared to controls. Scatter chart showing A. CD31 level in control and AD cases stratified to Braak tangle stage: BS0‐II (*n* = 17), III‐IV (*n* = 23), V‐VI (*n* = 21) and B. CD31 level in controls (n = 21), AD (*n* = 40), and VaD (*n* = 18). Each dot represents an individual case. Mean with SEM bars are shown for each graph. One‐way ANOVA following Tukey's post hoc comparison test.
**Figure S4.** Biochemical evidence of cerebral hypoperfusion and blood–brain barrier (BBB) leakiness within the parietal cortex. Scatter chart showing A. MAG:PLP1 ratio stratified to Braak tangle stage: BS0‐II (*n* = 17), III‐IV (*n* = 23), V‐VI (*n* = 21); B. Fibrinogen levels stratified to Braak tangle stage: BS0‐II (n = 17), III‐IV (n = 23), BSV‐VI (*n* = 22); C. MAG:PLP1 ratio stratified to control (*n* = 20), AD (*n* = 41), VaD (*n* = 18); and D. Fibrinogen stratified to control (n = 20), AD (*n* = 42), VaD (n = 18). Each dot represents an individual case. Mean with SEM bars are shown for each graph. One‐way ANOVA following Tukey's post hoc comparison test (A, B and D) and Kruskal‐Wallis test with Dunn's post hoc comparison test (C). * *p* < 0.05 **** *p* < 0.0001.
**Table S1.** Detailed summary of cases with UN Brain bank Unique IDs.

## Data Availability

The data that support the findings of this study are available from the corresponding author upon reasonable request.
