# Supplementary material for: Dysregulated TIE‐2 expression is associated with blood‐brain barrier leakiness and Alzheimer's disease‐related neuropathology
Source: Brain Pathol. 2026 Jun 7;36(6):e70109. doi: 10.1111/bpa.70109 (PMC13630861; doi:10.1111/bpa.70109)
Supplement: Supplementary file 1 [file BPA-36-e70109-s001.docx]

**Supplementary material**

(A) **Cognitively normal**

(B) **Mild-cognitively impaired**

**(C) Alzheimer’s disease**

**Supplementary Figure 1.** Heatmaps showing correlations between CSF TIE-1 and TIE-2 in relation to markers of ADNC, neuronal injury, and neuroinflammation. Data from Cohort (ii).

**Supplementary Figure 2.** Heatmap showing correlations between sTIE-1, sTIE-2, and ANGPT2. Data from Cohort (ii).

**Supplementary Figure 3.** MVF CD31 levels are unaltered in relation to Braak tangle stage (BS) and do not differ in AD or VaD compared to controls. Scatter chart showing **A.** CD31 level in control and AD cases stratified to Braak tangle stage: BS0-II (n = 17), III-IV (n = 23), V-VI (n = 21) and **B.** CD31 level in controls (n = 21), AD (n = 40), and VaD (n = 18). Each dot represents an individual case. Mean with SEM bars are shown for each graph. One-way ANOVA following Tukey’s post hoc comparison test.

**Supplementary Figure 4.** Biochemical evidence of cerebral hypoperfusion and blood-brain barrier (BBB) leakiness within the parietal cortex. Scatter chart showing **A.** MAG:PLP1 ratio stratified to Braak tangle stage: BS0-II (n = 17), III-IV (n = 23), V-VI (n = 21); **B.** Fibrinogen levels stratified to Braak tangle stage: BS0-II (n = 17), III-IV (n = 23), BSV-VI (n = 22); **C.** MAG:PLP1 ratio stratified to control (n = 20), AD (n = 41), VaD (n = 18); and D. Fibrinogen stratified to control (n = 20), AD (n = 42), VaD (n = 18). Each dot represents an individual case. Mean with SEM bars are shown for each graph. One-way ANOVA following Tukey’s post hoc comparison test (A, B and D) and Kruskal-Wallis test with Dunn’s post hoc comparison test (C). * P < 0.05 **** P < 0.0001

**Supplementary Table 1: Detailed summary of cases with UN Brain bank Unique IDs**

| **UKBBN ID** | **Age** | **Sex** | **PM**  **delay** | **Diagnosis** | **Braak-tangle stage** | **Cognitive status** |
| --- | --- | --- | --- | --- | --- | --- |
|  |  |  |  |  |  |  |
| 8642 | 84 | F | 60 | VaD | BS III-IV | CI |
| 8662 | 81 | M | 66 | VaD | BS 0-II | CI |
| 8669 | 93 | M | 30 | VaD | BS III-IV | CI |
| 8675 | 83 | M | 24 | VaD | BS 0-II | CI |
| 8706 | 72 | M | 42 | Con | BS 0-II | NO CI |
| 8708 | 90 | M | 45 | Con | BS 0-II | NO CI |
| 8723 | 80 | M | 67 | Con | BS III-IV | NO CI |
| 8739 | 93 | F | 18 | Con | BS 0-II | NO CI |
| 8751 | 82 | M | 30 | Con | BS 0-II | NO CI |
| 8779 | 69 | M | 66 | Con | BS 0-II | NO CI |
| 8787 | 77 | F | 85 | VaD | BS 0-II | CI |
| 8799 | 90 | F | 31 | VaD | BS 0-II | CI |
| 8848 | 77 | F | 43 | AD | BS III-IV | CI |
| 8861 | 89 | M | 30 | VaD | BS III-IV | CI |
| 8927 | 67 | M | 54 | VaD | BS III-IV | CI |
| 8949 | 79 | M | 24 | Con | - | NO CI |
| 8952 | 84 | M | 30 | VaD | BS 0-II | CI |
| 8975 | 76 | M | 40 | VaD | BS III-IV | CI |
| 8987 | 88 | F | 84 | AD | BS III-IV | CI |
| 8997 | 74 | F | 12 | AD | BS V-VI | CI |
| 9026 | 79 | M | 28 | AD | BS V-VI | CI |
| 9027 | 89 | M | 18 | AD | BS III-IV | CI |
| 9031 | 85 | M | 66 | AD | BS V-VI | CI |
| 9038 | 82 | F | 96 | AD | BS III-IV | NO CI |
| 9076 | 84 | F | 20 | AD | BS V-VI | CI |
| 9086 | 77 | M | 10 | AD | BS III-IV | NO CI |
| 9093 | 80 | M | 50 | AD | BS V-VI | CI |
| 9108 | 79 | M | 56 | VaD | BS 0-II | CI |
| 9112 | 74 | F | 52.5 | AD | BS V-VI | CI |
| 9122 | 83 | F | 5 | AD | BS V-VI | CI |
| 9123 | 74 | F | 35 | AD | BS V-VI | CI |
| 9136 | 77 | F | 26 | AD | BS V-VI | CI |
| 9154 | 81 | M | 28 | AD | BS V-VI | CI |
| 9162 | 63 | M | 43 | AD | BS V-VI | CI |
| 9173 | 86 | F | 31 | AD | BS V-VI | CI |
| 9174 | 77 | M | 37 | AD | BS III-IV | CI |
| 9192 | 79 | M | 77 | VaD | BS 0-II | CI |
| 9198 | 77 | F | 14 | AD | BS V-VI | CI |
| 9205 | 85 | F | 85 | AD | BS V-VI | CI |
| 9224 | 89 | F | 22 | VaD | BS 0-II | CI |
| 9261 | 83 | M | 48 | AD | BS V-VI | CI |
| 9262 | 81 | M | 4 | AD | BS V-VI | CI |
| 9266 | 80 | M | 72 | AD | BS V-VI | CI |
| 9269 | 83 | M | 99 | AD | BS III-IV | CI |
| 9274 | 78 | M | 49 | AD | BS V-VI | CI |
| 9299 | 90 | M | 5.5 | Con | BS 0-II | NO CI |
| 9303 | 69 | M | 12 | AD | BS V-VI | CI |
| 9311 | 93 | M | 37.75 | Con | BS III-IV | NO CI |
| 9315 | 67 | F | 24.25 | AD | BS V-VI | CI |
| 9324 | 95 | M | 21 | AD | BS III-IV | CI |
| 9327 | 92 | F | 46 | AD | BS III-IV | CI |
| 9329 | 80 | M | 45.75 | Con | BS 0-II | NO CI |
| 9338 | 95 | M | 27 | AD | BS III-IV | CI |
| 9340 | 94 | F | 21 | Con | BS 0-II | NO CI |
| 9343 | 80 | M | 24 | AD | BS III-IV | CI |
| 9344 | 92 | M | 34.25 | Con | BS 0-II | NO CI |
| 4205 | 87 | M | 24 | Con | BS 0-II | NO CI |
| 4208 | 78 | F | 54 | VaD | BS 0-II | CI |
| 4212 | 89 | F | 46.5 | AD | BS III-IV | NO CI |
| 4215 | 80 | F | 26 | AD | BS III-IV | CI |
| 4216 | 84 | F | 48 | AD | BS III-IV | CI |
| 4220 | 86 | F | 13.5 | AD | BS III-IV | CI |
| 4223 | 84 | F | 17.5 | VaD | BS 0-II | CI |
| 9361 | 83 | M | 11 | AD | BS V-VI | CI |
| 4229 | 87 | F | 47 | Con | BS III-IV | NO CI |
| 4236 | 74 | M | 23.5 | AD | BS III-IV | CI |
| 9376 | 83 | F | 66 | AD | BS III-IV | CI |
| 9387 | 90 | M | 45 | VaD | BS 0-II | CI |
| 9389 | 68 | F | 38.75 | Con | BS 0-II | NO CI |
| 9395 | 80 | M | 62.5 | AD | BS V-VI | CI |
| 9397 | 97 | F | 36 | AD | BS III-IV | CI |
| 9433 | 88 | M | 36 | AD | BS III-IV | CI |
| 14403 | 68 | F | 21.75 | VaD | BS 0-II | CI |
| 19608 | 96 | M | 21 | Con | BS 0-II | NO CI |
| 19628 | 76 | F | 50 | VaD | BS 0-II | CI |
| 24337 | 92 | M | 56.5 | Con | BS 0-II | NO CI |
| 24308 | 80 | M | 50 | AD | BS III-IV | NO CI |
| 24311 | 82 | F | 36 | Con | BS 0-II | NO CI |
| 6.30186 | 69 | F | 38.25 | Con | BS 0-II | NO CI |
| 6.31516 | 94 | F | 43.25 | Con | BS 0-II | NO CI |
| 6.32578 | 86 | F | 36.25 | Con | BS 0-II | NO CI |

BB No: Brain bank number, F: female, M: male, PM delay: post-mortem delay, EAD: early-stage AD or Alzheimer’s changes, CI: cognitive impairment.
